# Transaminase abnormalities and adaptations of the liver lobule manifest at specific cut-offs of steatosis

**DOI:** 10.1038/srep40977

**Published:** 2017-01-20

**Authors:** Andrew Hall, Claudia Covelli, Roberta Manuguerra, Tu Vinh Luong, Elena Buzzetti, Emmanuel Tsochatzis, Massimo Pinzani, Amar Paul Dhillon

**Affiliations:** 1 The Department of Cellular Pathology, Royal Free London NHS Foundation Trust, London, NW3 2QG, UK; 2 Institute for Liver and Digestive Health, UCL, Royal Free Campus, London, NW3 2QG, UK; 3 The Department of Cellular Pathology, UCL Medical School, Royal Free Campus, London, NW3 2QG, UK

## Abstract

There is little documented evidence suggesting that liver fat is responsible for liver injury in the absence of other disease processes. We investigated the relationships between liver fat, aminotransferases and hepatic architecture in liver biopsies with simple steatosis. We identified 136 biopsies with simple steatosis from the Royal Free Hospital Archives with both clinical data and sufficient material. Digital image analysis was employed to measure fat proportionate area (mFPA). Hepatocyte area (HA) and lobule radius (LR) were also measured. There were significant increases in ALT (p < 0.001) and AST (p = 0.013) with increased fat content and evidence to suggest both 5% and 20% mFPA as a cut-off for raised ALT. In liver with increased fat content there were significant increases in HA (p < 0.001). LR also increased as mFPA increased to 10% (p < 0.001), at which point the lobule ceased to expand further and was counterbalanced with a decrease in the number of hepatocytes per lobule (p = 0.029). Consequently there are mechanisms of adaption in the liver architecture to accommodate the accumulation of fat and these are accompanied by significant increases in transaminases. These results support the generally accepted cut-off of 5% fat for steatosis and indicate 20% as a threshold of more severe liver injury.

Non-alcoholic fatty liver disease (NAFLD) has a prevalence of 20–30% in developed countries^1^ and is a common cause of elevated liver enzymes^2^. The histology of NAFLD has a wide spectrum from simple steatosis to steatohepatitis with or without fibrosis and cirrhosis^3^,^4^. The presence of fat is an important element of the disease and can lead to inflammation and fibrosis^5^,^6^,^7^. Along these lines, liver fat content contributes 3 points out of 8 in the NAFLD activity score (NAS)^4^. Considering that a certain degree of fatty hepatocellular infiltration is a frequent finding^8^,^9^ and that, in general, the presence of fatty liver is not necessarily associated with inflammation and fibrosis, a legitimate question that would be important to answer is: how much liver fat is too much? Based on what is reported in the literature, the answer to this question appears problematic. A Swiss study showed that healthy non-obese individuals have 3.05–5.25% liver fat dependent on age and sex when measured using magnetic resonance imaging (MRI). An American study performed in 2004, using magnetic resonance spectroscopy (MRS), found that 5.56% fat corresponded to the 95^th^ percentile and this parameter was proposed as a cut-off for the presence of an abnormal liver fat quantity, which is termed steatosis^10^. The complexity tends to increase considering a wider age-range. Accordingly, it has been found that in non-obese children the median fat fraction was 1.6%-2.7% up to a maximum of 6.5–8.0% dependent on whether measured using dual or triple echo gradient-recalled echo sequence MRI^11^. Another study performed on obese children without NAFLD found a mean hepatic fat fraction of 2.0% up to a maximum of 4.69%^12^. MRI spectroscopic fat fraction in histological graded normal to mild hepatic steatosis is 0–13%^13^.

In spite of this lack of a precise definition, liver steatosis is in general defined as >5% fat, and the presence of steatosis is considered the most common explanation for abnormal liver function tests (LFT) in the absence of other apparent causes of acute or chronic liver injury. Increased LFTs have previously been correlated with steatosis using MRI in obese patients^14^ and using grading systems in NAFLD^15^. A significant increase in alanine aminotransferase (ALT) has been reported for individuals with simple steatosis (5% fat assessed microscopically) compared to those with normal liver histology^16^. However, there is little other evidence to sustain that 5% fat represents a reliable cut-off to justify the presence of abnormal LFTs.

In summary, there is a need for a more precise definition of steatosis in order to provide a more accurate clinical framing of the presence of abnormal LFTs in the absence of other apparent causes of liver injury. To answer the question if fat and LFTs are related it is mandatory to, a) select cases without an evident cause of liver injury and characterised as histologically confirmed simple steatosis, and b) employ an accurate and validated quantitative measure for hepatic fat.

The present study was specifically designed to provide a more clinically relevant definition of simple steatosis. To this aim, we investigated the relationships between liver fat content, LFTs and liver architecture in liver biopsies that were either normal or characterized by simple steatosis. To address this task we employed digital image analysis (DIA) to measure liver fat from haematoxylin and eosin (H&E) stained biopsy sections. The DIA of measured fat proportionate area, (mFPA) is the area of a histological section of liver biopsy that is occupied by fat as a proportion of the whole parenchyma area. By using this methodology we have previously reported a degree of accuracy and repeatability in histological liver fat assessment which is much higher than the traditional subjective method^17^,^18^. In addition, we made a special effort in measuring the morphological abnormalities of both hepatocytes and the liver lobule typical of steatotic liver tissue.

## Methods

In order to identify cases with simple steatosis a database search was conducted for all histology specimens coded as liver and fatty between 2000 and 2014 in the Royal Free Hospital archives. To exclude livers with a known aetiology associated with fatty liver such as alcoholic liver disease (ALD) and other possible influencing factors such as HIV and diabetes, we conducted 1) a histology database review (EB, CC), 2) full clinical review of patient notes (EB), 3) histology slide review (RM). We identified 205 liver biopsies from patients with normal or fatty livers with no other chronic liver disease. Of these, 161 had clinical biochemistry LFT tests [ALT, aspartate aminotranserase (AST), alkanine phosphatase (ALP), bilirubin, albumin, gamma glutamyl transferase, (GGT)] completed within two weeks prior to the biopsy. One hundred and fifty one had sufficient biopsy material for mFPA analysis. There were 136 biopsies with both LFTs and sufficient material for mFPA and hepatocyte/lobule measurements. All methods were carried out in accordance with relevant guidelines and regulations, experimental protocols were approved by the Royal Free Hospital Ethics Committee (07/Q0501/50) and informed consent was obtained.

### Digital image analysis of mFPA

Liver fat was measured using digital images of 4 μm thick sections stained with H&E. The DIA was performed as reported previously^18^. In brief, the DIA used a visual basic script for Zeiss Axiovision (version 4.8.2.) in which binary segmentation of red/green/blue colour channels was used to distinguish cytoplasm from fat vacuoles. The mFPA was calculated as the area occupied by the fat as a proportion of the area of the whole parenchyma. An editing step was included and, where practicable, dilated sinusoids, blood vessels, fibrous areas and confounding artifacts were manually edited and excluded from analysis. To assess the mFPA of a biopsy, 21 randomly selected non-overlapping areas were imaged at x20 objective magnification. Details of the procedure followed to select the sample size of 21 images are provided in the Supplementary Information.

### Hepatocyte Area (HA)

Fifty randomly selected hepatocytes were chosen from each biopsy and were measured in 2 perpendicular dimensions, (from each biopsy 5 hepatocytes were randomly selected from each of 10 random selected images). The areas of the hepatocytes were calculated as an oval and hepatocyte areas (HA) are reported as the median of all 50 hepatocyte areas for each biopsy. The protocol for determination of sample adequacy for hepatocytes size measurements is detailed in the Supplementary Information.

### Lobule Radius (LR)

Consecutive sections stained with H&E, picro-Sirius Red^19^ and Gordon and Sweets reticulin stain^20^ were used to identify central veins. All portal tracts around the central vein that formed the pattern of a lobule were located and the portal tract to central vein distances were measured. The lobule radius (LR) is reported as the median of all the measurements for each biopsy.

### Hepatocytes per Lobule (HpL)

Using H&E stained sections it is not possible to calculate the number of hepatocytes per lobule as there is no easily distinguishable separation between each lobule. We considered the lobule to be hexagonal and we used the portal tract to central vein measurement as the radius of the hexagon. With these assumptions made:


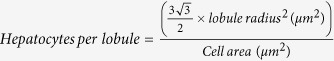
The number of hepatocytes per lobule (HpL) is not the actual number of hepatocytes rather an approximation.

### Statistics

The data were predominantly non-parametric. Numerical variables were summarized as medians, upper/lower quartiles and ranges. Relationships between continuous variables were assessed using Spearman rank correlation coefficient. Differences between independent distributions were assessed using the Mann-Whitney U test for 2 categories and Kruskal Wallis test for 3 or more. Significance testing was two-sided and the type 1 error was 0.05. All statistics were performed using SPSS (version 22.0; SPSS Inc, Chicago, IL).

## Results

Demographic data, liver biochemistry and lobule architecture measurements are summarised in Table 1. The demographics of the cohort itself were largely unremarkable. There were 78 males and 58 females and we found that there was significantly higher mFPA in the male population than in the female (p = 0.035), this was as expected^21^.There were significant differences between genders in the distribution of age (p = 0.001), ALT (p = 0.038), GGT (p = 0.036), bilirubin (p < 0.001), albumin (p < 0.001) and ALP (p < 0.001).

Table 2 shows the correlations between mFPA and ALT, AST, HA, LR and HpL. mFPA correlated significantly well with HA (R_s_ = 0.821, P < 0.001) and LR (R_s_ = 0.637, p < 0.001) and weakly with ALT (R_s_ = 0.408, p < 0.001), AST (R_s_ = 0.233, p = 0.04) and HpL (R_s_ = −0.266, p = 0.019). All variables, morphometric and biochemical, were tested for correlation with no additional significant correlations found.

**Figure 1:**
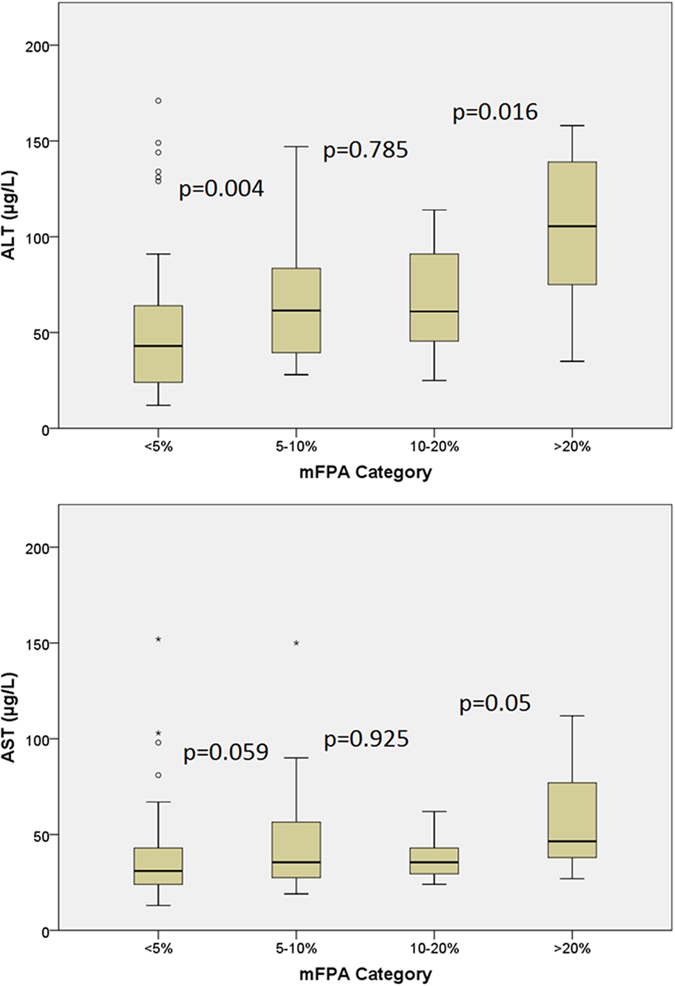
A box plot showing the distribution of ALT and AST when the population is split into increments of mFPA. The box of the box plot illustrates the median, upper and lower quartiles for each population of <5%, 5–10, 10–20% and >20% mFPA. The attached bars indicate the range and the discrete points are the outliers. The probability that two populations are drawn from the same population (Mann-Whitney test) are illustrated on the diagram between each pair of populations, e.g. there is a statistical difference in ALT between <5% mFPA and 5–10% mFPA because p = 0.004.

To address one of the main questions at the origin of the present work, we initially tested the data to determine if there were significant differences in ALT or AST as mFPA increases. We found that there were significant differences in ALT and AST when using a cut-off of 5% (ALT p < 0.001 and AST p = 0.005), 10% (ALT p = 0.001 and AST p = 0.027) and 20% mFPA (ALT p < 0.001 and AST p = 0.013). Therefore we broke mFPA down into brackets of <5%, 5–9.9%, 10–19.9% > 20% to investigate any changes in ALT and AST in more detail. Figure 1 demonstrates that the increase in mFPA was associated with parallel increases of ALT and AST. There was an overall significant difference in ALT and AST across the mFPA categories (p < 0.001, p = 0.013 respectively). There were also a significant difference in between some of the individual mFPA categories for ALT (between < 5% and 5–9.9% p = 0.004, 10–19.9% and > 20% p = 0.016) and AST (between 10–19.9% and > 20% p = 0.05). These are graphically represented in Fig. 1.

We next investigated the effect of mFPA on the other LFTs, namely GGT, bilirubin, albumin and ALP. There was a significant reduction in GGT for biopsies with > 5% mFPA compared to < 5% mFPA (p = 0.020). There were no significant changes in bilirubin, albumin or ALP at 5%, 10% or 20% mFPA thresholds. When the data were broken down into the mFPA brackets of <5%, 5–9.9%, 10–19.9% > 20% there were no significant differences between any adjacent pairs of categories. The box plots are available in the Supplementary information.

We found that the size of hepatocytes and lobules increased in samples with higher mFPA. HA and LR increased significantly when using a cut-off of 5%, 10% or 20% mFPA (all p < 0.001) and there was a significantly lower HpL using a cut-off of 10% and 20% mFPA (p = 0.02 and p = 0.014 respectively).

**Figure 2:**
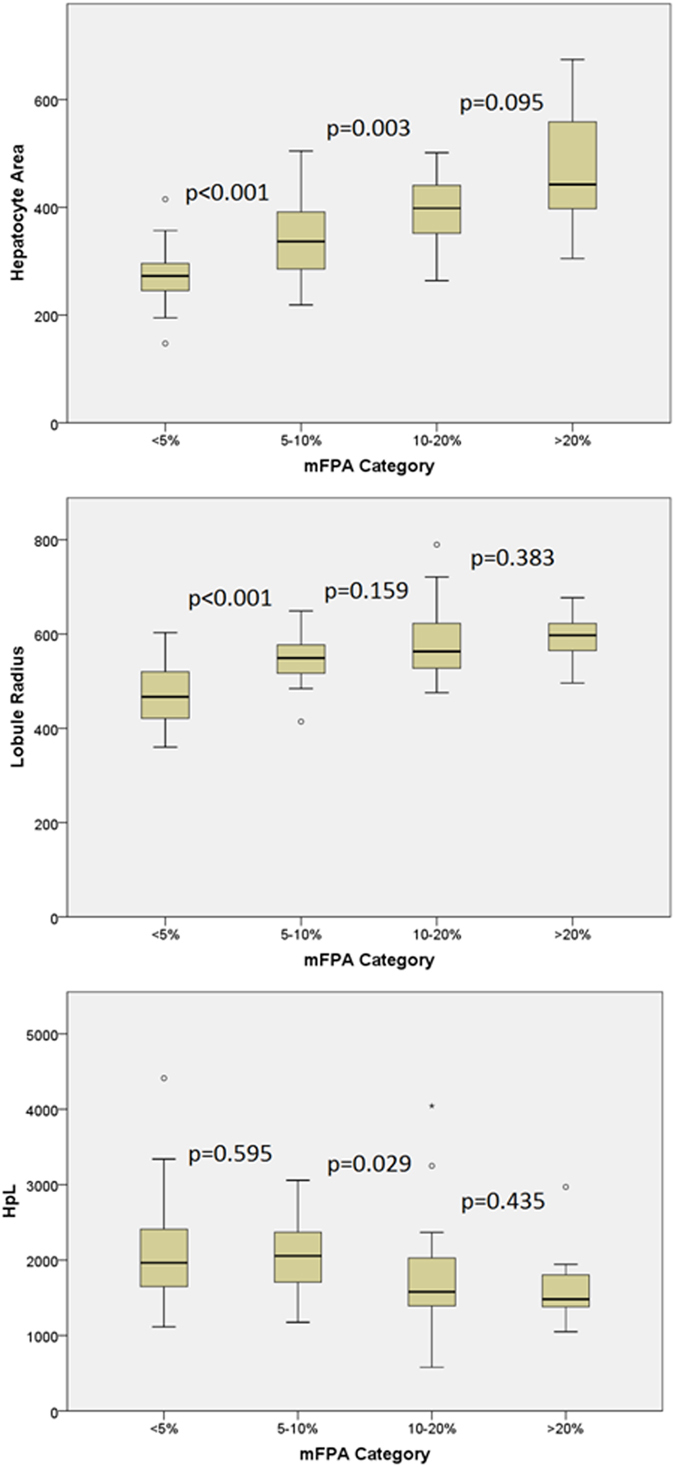
A box plot showing the distribution of hepatocyte area, lobule radius and the number of hepatocytes per lobule when the population is split into increments of mFPA. The box of the box plot illustrates the median, upper and lower quartiles for each population of <5%, 5–10, 10–20% and >20% mFPA. The attached bars indicate the range and the discrete points are the outliers. The probability that two populations are drawn from the same population (Mann-Whitney test) are illustrated on the diagram between each pair of populations, e.g. there is a statistical difference in HA between <5% mFPA and 5–10% mFPA because p < 0.001.

The HA, LR and HpL were further investigated using the previously described <5%, 5–9.9%, 10–19.9% > 20% brackets. There was a statistically significant increase in both HA (p < 0.001) and LR (p < 0.01) and a reduction in HpL (p = 0.012) as mFPA increased. These are graphically represented in Fig. 2 and the measurement values given in Table 3.

**Figure 3:**
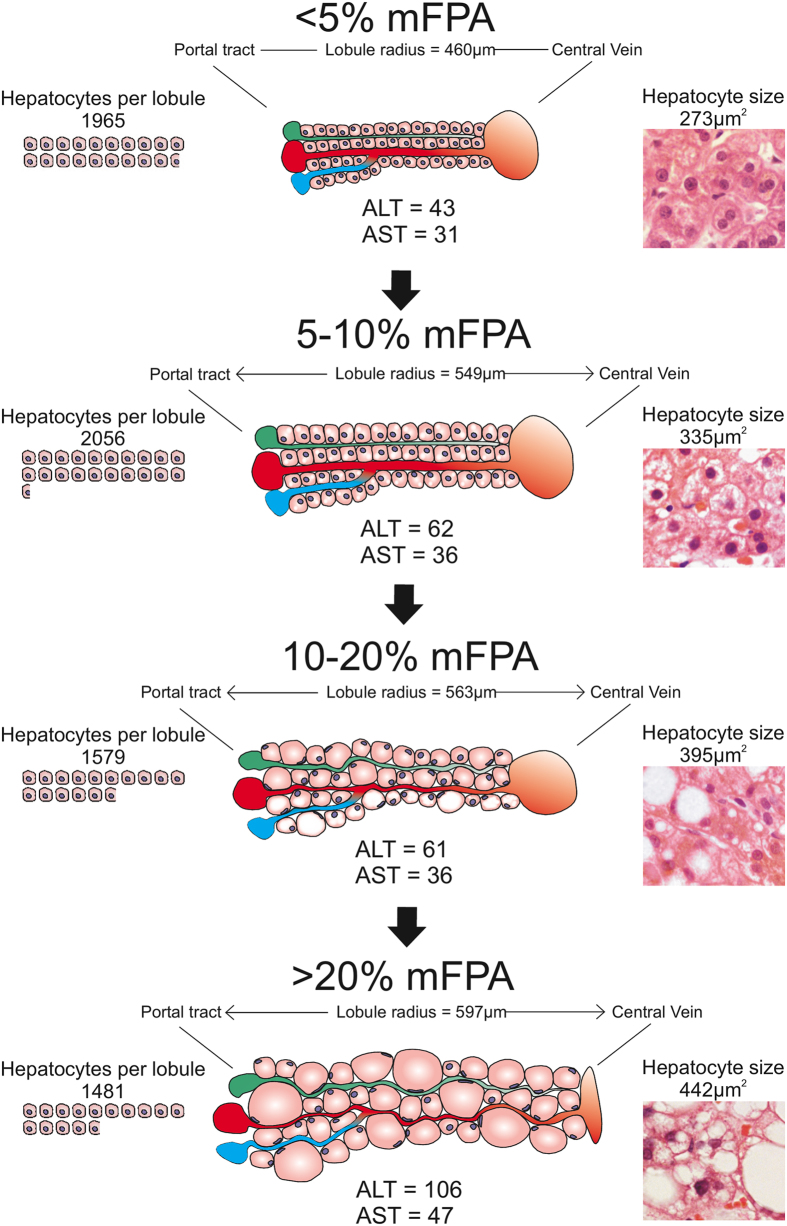
Diagrammatic representations of the changes in the lobule with increasing fat content. As the liver accumulates fat in the hepatocytes (5–10% mFPA) they swell in size and the lobule initially similarly expands. The number of hepatocytes in the lobule remains consistent but there is a significant rise in both ALT and AST. As the hepatocytes accumulate more fat (10–20% mFPA) the lobule does not significantly increase in size but there is a corresponding reduction in the number of hepatocytes. Further accumulation of fat by the liver (>20% mFPA) produces even larger hepatocytes and a further incremental increase in ALT and AST. The cases with >20% mFPA showed architectural disorganization at the lobular level with a notably compressed central vein often causing difficulties in identifying it on H&E.

HA was significantly higher with each increase in mFPA category up to 20% mFPA (p < 0.001 and p = 0.003). As mFPA increased from < 5% to 5–9.9% the LR increased (p < 0.001), However, further increase in mFPA beyond 10% did not cause a significant increased in LR. The only significant change in the number of HpL also occurred with a reduction in HpL at 10% mFPA (p = 0.029); this coincides with the continued increase in HA and the cessation of LR expansion at 10% mFPA. Over 20% mFPA the hepatocytes and lobules were increasingly disorganised and consequently difficult to assess. The changes in hepatocyte and lobule parameters are better demonstrated in Figs 2 and 3.

**Figure 4:**
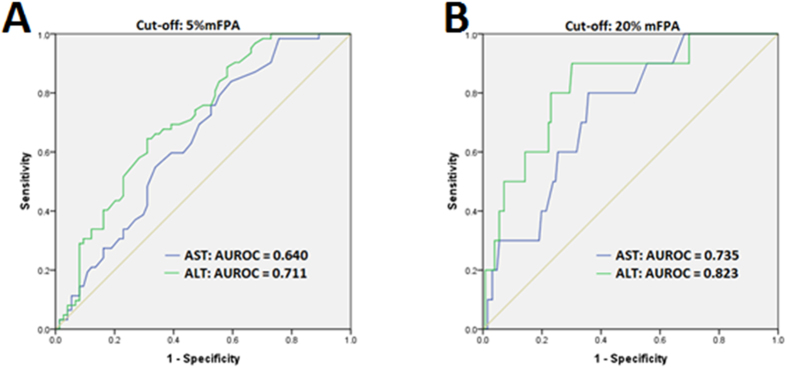
ROC curves showing AST and ALT used as a cut-off for 5% and 20% mFPA. As a test for a 20% cut off of mFPA, AST is a ‘fair test’ (AUROC = 0.735) and ALT is ‘good’ (AUROC = 0.823). As a test for 20% mFPA an ALT cut-off of 67 U/L gives a sensitivity of 90% and specificity of 70% and a cutoff of 75 U/L gives a sensitivity of 80% and specificity of 77%.

As we had found a significant increase in transaminases at 5% and 20% mFPA we constructed receiver operating characteristic (ROC) curves for AST and ALT as predictors of mFPA using these as cut-offs, Fig. 4. As a test for > 5% mFPA, AST is a ‘poor’ test (area under ROC, (AUROC) = 0.64) and ALT is only slightly better (AUROC = 0.71). Using an AST cut-off of 31 U/L (upper normal limit) as a test for 5% mFPA gives a sensitivity of 70% and specificity of 50%, and an ALT cut-off of 33 U/L (upper normal limit) gives a sensitivity of 90% and specificity of 40%.

Using ALT and AST as a test for a 20% cut off of mFPA, AST is a ‘fair test’ (AUROC = 0.74) and ALT is ‘good’ (AUROC = 0.82). An AST cut-off of 31 U/L (upper normal limit) as a test for 20% mFPA gives a sensitivity of 90% and specificity of 45% and 41 U/L gives a sensitivity of 70% and specificity of 67%. An ALT cut-off of 33 U/L (upper normal limit) gives a sensitivity of 100% and specificity of 28%. The best compromise between sensitivity and specificity for ALT is at 67 U/L (90% and 70%) to 75 U/L (80% and 77%).

## Discussion

This study is the first to show that in livers with simple steatosis the amount of hepatic fat is statistically correlated with the elevation of ALT and AST (R_s_ = 0.358, p < 0.001 and R_s_ = 0.139, p = 0.021 respectively). This study also shows that there are consistent changes in hepatocyte and lobule size with increased quantities of liver fat that may represent the pathophysiological background of LFT abnormalities. Along these lines, both ALT and AST show significant changes as mFPA increases from <5% to >5%, then show no significant change until >20% mFPA when a further increase in ALT and AST is observed. Overall, our observations support the clinical guidelines of 5% cut-off for defining steatosis and its possible association with abnormal LFTs in the absence of other causes of liver injury.

It is a common clinical observation that ALT and AST levels are often moderately increased in patients with fatty liver, characterized by fluctuations depending on dietary changes and tend to improve following even moderate weight loss together with a decrease in hepatic fatty infiltration^22^. Moreover, serum ALT levels appears to be related to liver fat content, insulin resistance and type 2 diabete^14^,^15^,^23^,^24^,^25^,^26^. From the pathophysiological point of view, excessive cytoplasm fat deposits in the hepatocyte have been implicated as a direct cause of cell damage^27^. However, how this correlates with increased ALT and AST levels still represents a matter of speculation. It is important to note that ALT is present in two isoforms, ALT 1 and ALT 2. As a cytoplasmic protein, ALT1 is relatively easier to “leak” out of the cell than the prevalently mitochondrial ALT2. In this regard, elevation of ALT2 in serum in conjunction with ALT1 elevation may be a better indicator of “true” liver damage with mitochondria involvement. On the other hand, the current routine enzyme activity assay is optimized for measuring ALT1 activity because it represents the prevailing form of ALT in the serum^28^.

Accordingly, the commonly observed increased ALT levels are likely a sign of the membrane leaking cytoplasmic enzymes. On the contrary increased AST, that shows changes to a lesser extent than ALT, is more likely a sign of mitochondrial stress and in general indicate a worse and more chronic cellular stress. On the other hand, abnormal ALT levels as those reported in the present study are relative to a situation of fat accumulation without any clear evidence of oxidative-stress related inflammation and necrosis that would have led to more substantial ALT and AST changes. Similar considerations can be made for the abnormalities in GGT levels. The small changes of in GGT between <5% and >5% do not have an obvious explanation but tends to exclude the impact of any alcohol excess in the cases presenting with higher FPA.

It is widely established that liver steatosis, defined as a pathologist’s estimate of >5% fat, can be a potential explanation of abnormal LFTs in the absence of any other apparent cause of liver injury. 5% fat is also often considered the cut-off between normal and steatotic livers^3^,^4^,^15^. Our previous work demonstrated that 5% fat as estimated by expert pathologists is approximately equal to 5% mFPA^17^,^18^. The results of this study suggests that in livers with simple steatosis, the biopsies with >5% mFPA are associated with an increased AST but the rise in the median is only small (AST: 31 to 37) and as a test is considered poor (i.e. AUROC = 0.64). For ALT, the rise in activity between <5% and >5% (43 to 65) suggests a higher cut-off than what is usually considered the normal/elevated level and as a test it is poor/fair (i.e. AUROC = 0.71). In the context of simple steatosis 5% fat appears to be necessary but not sufficient, for abnormal LFTs.

The next point that we aimed to clarify was the relationship between liver fat content and hepatocytes size since there is no available reference in the literature. Hepatocytes diameter is commonly reported to be around 30 μm: 30–40 μm^29^; 30 μm^30^; 20–30 μm^31^. One of the few studies in which the size was actually measured reported an average diameter of 15.9–19.1 μm^32^. Some studies have measured hepatocyte volumes and if calculated from the volumetric data, assuming that the hepatocyte is spheroid, they found the hepatocyte diameters to be 27.8 μm^33^, 17.6 μm^34^ and 19.5–21.2 μm^35^. Hepatocyte size in humans correlates with portal hypertension in alcoholic liver diseases both with and without the presence of cirrhosis^36^,^37^,^38^, although this has been disputed^39^. Hepatocyte size has also been shown to be larger in cirrhotic than non-cirrhotic livers^40^. Finally, it is well accepted that “hepatocyte volume change (swelling) is part of normal physiology…”^29^, although the size of hepatocytes in direct relationship with steatosis has not yet been studied.

Similar considerations can be made for the dimension of the liver lobule. It is almost 200 years since Kiernan stated “the form of the lobules will be now easily understood; their dimensions are known to all anatomists”^41^. Kiernan did not state the lobule dimensions. The human liver lobule is said to have a diameter of; 1 mm^42^, 1–1.3 mm^43^, 1–2 mm^44^ with a height of 0.3–0.9 mm and volume 0.1–0.9 mm^3^ 45. Studies in animals show that the size of the liver lobule in rats and sheep is not a constant and can change in size considerably under different conditions^46^,^47^,^48^ though to our knowledge it has never been studied in humans.

Overall our observations concerning the median hepatocyte size (HA 281 μm^2^ or diameter of 18.9 μm) and lobule size (radius of 491 μm) in non steatotic livers are in agreement with those provided in the literature^29^,^30^,^31^,^32^,^33^,^34^,^35^,^36^,^37^,^38^,^39^,^40^. Relevantly, we found that as hepatocytes change from storing normal amounts of fat (<5%) to abnormal amounts (5–9.9%), they swell in size in order to accommodate the extra lipid content. An increase in the HA coincides with an expansion of the LR accommodating the larger hepatocytes and there is no significant loss in the number of hepatocytes from the lobule. As the liver becomes increasingly fatty, i.e. mFPA 5–9.9% to 10–19.9%, there is an additional increase in HA. However, the lobule does not significantly increase in size as there is a corresponding significant drop in the number of HpL. Additional increases in mFPA (>20%) were not associated with significant changes in HA, LR or HpL. Nonetheless this could be due to lower sample size (n = 10).

The liver therefore responds to increasing amounts of fat with architectural mechanisms of adaptation. Using DIA to measure mFPA we found a combination of important observations: 1) the liver lobule expands with increasingly larger hepatocytes as they accumulate fat; 2) above 10–20% mFPA the liver lobules cease to expand significantly further and the numbers of hepatocytes decreases; 3) ALT and AST levels are increased in liver with increased fat content and they show significant increases at 5% mFPA (hepatocytes begin to swell and lobules expand) and 20% (after a significant reduction in the number of hepatocytes from the lobule).

Accordingly, 10–20% mFPA appears to be an important clinic-pathological turning point in “simple” steatosis. The observed process of “spatial adaptation”, i.e. the decrease in the number of hepatocytes per lobule and/or the swelling of hepatocytes as mFPA increases above 10–20% mFPA could be somehow responsible for the coincident elevation of LFTs in simple steatosis. Currently, above 5% fat there is no risk stratification in simple steatosis i.e. 20% fat poses no increased risk than 10% fat. However, according to the results of this study, 20% mFPA could be considered an important cut-off and represent a borderline pathophysiological condition leading to further evolution of NAFLD, including the development of NASH. Accordingly, this level of fat accumulation, which is still within what is defined “simple steatosis”, should be considered the ideal clinico-pathological correlate for further studies aimed at elucidating the cellular and molecular mechanisms responsible for the transition from steatosis to steatohepatitis.

In conclusion the results presented herein suggest that in the absence of inflammation or other causes of liver damage there is a clear link between the amount of fat within the liver and the amount of ALT and AST activity in the blood. Furthermore there are significant mechanisms of adaption in the liver architecture to accommodate the fat. The data presented in this paper generally supports the clinical guidelines of 5% cut-off for abnormal steatosis and 20% mFPA as a warning threshold potentially predicting evolution towards more pronounced hepatocyte injury and NASH.

## Additional Information

**How to cite this article**: Hall, A. *et al*. Transaminase abnormalities and adaptations of the liver lobule manifest at specific cut-offs of steatosis. *Sci. Rep.* **7**, 40977; doi: 10.1038/srep40977 (2017).

**Publisher's note:** Springer Nature remains neutral with regard to jurisdictional claims in published maps and institutional affiliations.

## Supplementary Material

Supplementary Information

## Figures and Tables

**Table 1:** A table showing the median, lower quartile (LQ) and upper quartiles (UQ) of the demographic, liver biochemistry and lobule measurements data of cohort of patients.

	All Median (LQ-UQ)	Male Median (LQ-UQ)	Female Median (LQ-UQ)	<5%mFPA Median (LQ-UQ)	>5%mFPA Median (LQ-UQ)
**N**	136	78	58	74	62
Age^*^	46 (38–53)	41 (35–49)	53 (49–56)	50 (40–53)	43 (37–51)
mFPA^*¥^	5 (1.7–9.5)	7.0 (2.7–14.6)	3 (0.9–8.4)	1.4 (0.7–2.9)	9.3 (7.8–19.4)
ALT (U/L)^*¥^	60 (39–93)	64 (42–93)	54 (31–82)	51 (31–81)	68 (47–97)
**AST (U/L)**^**¥**^	35 (26–50)	36 (26–48)	35 (26–60)	34 (26–46)	37 (30–51)
GGT (U/L)^*¥^	78 (46–140)	64 (42–112)	104 (60–167)	107 (59–159)	66 (37–103)
Bilirubin (mmol/L)^*^	11 (7–15)	11 (8–16)	8 (6–11)	11 (7.5–12)	11 (7–15)
Albumin (g/L)^*^	47 (45–49)	48 (46–49)	45 (42–47)	46 (44–49)	47 (45–49)
ALP (U/L)^*^	82 (68–118)	73 (64–100)	105 (82–156)	100 (72–131)	78 (65–100)
**HA**^**¥**^	304 (263–371)	306 (260–391)	303 (276–332)	281 (250–303)	369 (305–413)
**LR**^**¥**^	520 (489–568)	520 (496–565)	525 (466–578)	491 (420–522)	564 (518–608)
**HpL**	1965 (1556–2422)	1941 (1551–2357)	1964 (1632–2548)	1973 (1582–2493)	1945 (1551–2357)

The lower quartile (LQ) and upper quartiles (UQ) are included in the parentheses. ^*^There is a significant difference in the between Male and Female populations (p < 0.05). ^¥^There is a significant difference between <5% mFPA and >5% mFPA populations (p < 0.05).

**Table 2:** A table of correlations of mFPA with transaminases and lobule measurements.

mFPA						
	Males		Females		Together	
	R_s_	P	R_s_	P	R_s_	P
ALT	0.408	P < 0.001	0.201	p = 0.130	0.358	P < 0.001
AST	0.233	p = 0.04	0.139	p = 0.298	0.198	P = 0.021
HA	0.821	p < 0.001	0.687	p < 0.001	0.816	p < 0.001
LR	0.637	p < 0.001	0.521	p = 0.001	0.64	p < 0.001
HpL	−0.266	P = 0.019	−0.252	P = 0.066	−0.219	p = 0.012

**Table 3:** A table showing the lobule measurements of our cohort of patients separated into categories of mFPA.

	mFPA < 5 Median (LQ-UQ)	mFPA 5–9.9 Median (LQ-UQ)	mFPA 10–19.9 Median (LQ-UQ)	mFPA 20–29.9 Median (LQ-UQ)
**n**	74	32	20	10
**m/f**	35/39	23/9	13/7	7/3
**Age** Years	50 (39–56)	44 (34–53)	45 (41–53)	51 (42–57)
**mFPA** %	1.1 (0.7–2.6)	7.5 (6.0–8.8)	16.2 (14.7–19.2)	23.8 (20.7–27.9)
**HA** μm^2^	273 (245–297)	335 (286–382)	395 (352–433)	442 (397–558)
**LR** μm	466 (421–520)	549 (516–577)	563 (527–623)	597 (565–623)
**HpL** hepatocytes	1965 (1650–2409)	2056 (1709–2367)	1579 (1392–2028)	1481 (1380–1804)

The lower quartile (LQ) and upper quartiles (UQ) are included in the parentheses.
