# Supplementary material for: Transaminase abnormalities and adaptations of the liver lobule manifest at specific cut-offs of steatosis
Source: Sci Rep. 2017 Jan 20;7:40977. doi: 10.1038/srep40977 (PMC5247698; doi:10.1038/srep40977)
Supplement: Supplementary Information [file srep40977-s1.pdf]

Supplementary Information: Transaminase abnormalities and adaptations of the liver lobule manifest at specific cut-offs of steatosis.

Authors: *Andrew Hall[1], Claudia Covelli[1], Roberta Manuguerra[1], Tu Vinh Luong[1], Elena Buzzetti [2], Emmanuel Tsochatzis[2], Massimo Pinzani[2], Amar Paul Dhillon[3].

Affiliations:

[1]*The Department of Cellular Pathology, Royal Free London NHS Foundation Trust, London, UK***,** NW3 2QG.

[2] *Institute for Liver and Digestive Health, UCL, Royal Free Campus, London, UK,* NW3 2QG.

[3]*The Department of Cellular Pathology, UCL Medical School, Royal Free Campus, London, UK***,** NW3 2QG.

Corresponding Author:

Andrew Hall,

Department of Cellular Pathology,

UCL Institute for Liver and Digestive Health,

Royal Free London NHS Foundation Trust,

London,

NW3 2QG,

UK

Tel: 0778 9992421

Fax: 0207 4353289

e-mail: andrewhall1@nhs.net

Method for the determination of an adequate sample size for hepatocyte size measurements:

We used H&E sections from 5 patients with a range of median hepatocyte areas, (199-457 μm^2). Non-overlapping images were taken at x20 objective magnification (OM) using a Zeiss Axioskop 40 with a Zeiss IcC5 camera in which the whole tissue area was sampled. The hepatocytes were measured in 2 dimensions. The first measurement was along the maximum diameter of the hepatocyte and the second the maximum diameter perpendicular to the first. All the hepatocytes in the biopsy were measured if nucleus and the cell membranes were identifiable. Hepatocyte area was calculated as an ellipse from the 2 diameter measurements.

$$Cell\ area\ (\mu m^2) = \pi \times \left(\frac{max\ lobule\ diameter}{2}\right) \times \left(\frac{perpendicular\ lobule\ diameter}{2}\right)$$

At least 2000 hepatocytes were measured per biopsy. A custom written MATLAB script was written that randomly selected a number of hepatocyte measurements and compared their average area to a reference value emulating and improving on previous work we have done on sample size calculation [1]. The reference value for the biopsy was the median hepatocyte area for the entire biopsy (median hepatocyte area for the 5 biopsies were 199, 216, 236, 299 and 457μm^2). In brief, the script would, for example, start by randomly (without replacement) selecting 2 hepatocytes from the 2000 and comparing the mean of their areas to the reference value and record the amount by which it deviated from the reference. The script repeated this random selection process 1000 times for each sample size from 1 to 2000 hepatocytes. In total, for all 5 biopsies, the script calculated 2 x10^6 random samples for each biopsy, in total 10x10^6 samples. The plots below show that a sample size of

approximately 50 HA measurements taken randomly from throughout the biopsy is representative of average hepatocyte size for a biopsy.

Supplementary Figure S1. A scatter plot showing the proportion of samples that were 95% accurate (i.e. deviated by less than 5% of the reference value) for each of the sample sizes from 1-2000 cells measured.

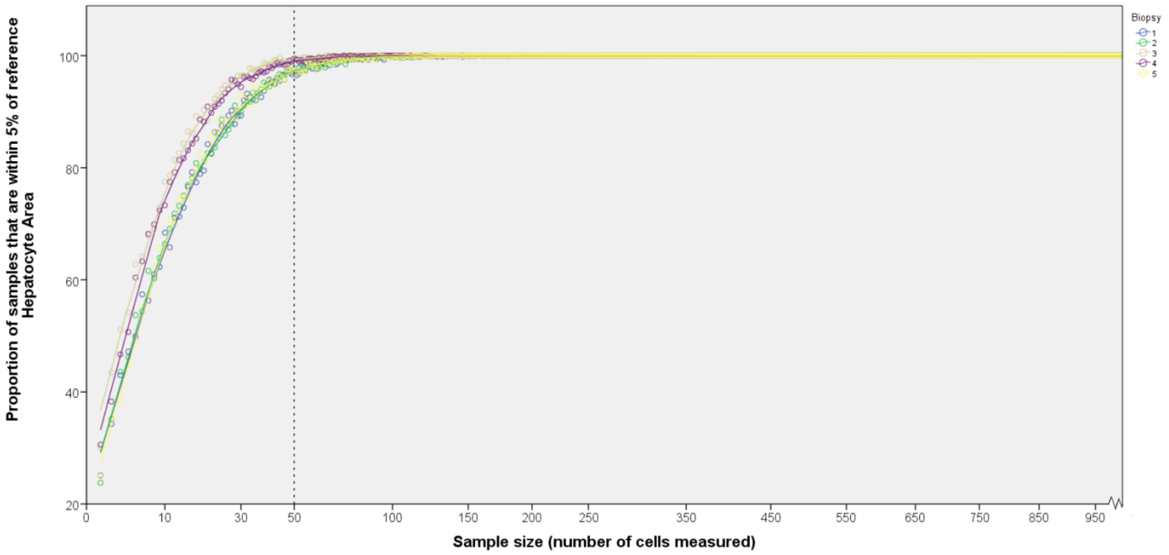

97-99.6% of samples were 95% accurate (i.e. deviated by less than 5% of the reference value) when a sample size of 50 hepatocytes was randomly selected from the each biopsy. Therefore to establish a protocol for average hepatocyte size we randomly selected 50 hepatocytes from each biopsy and calculated their median hepatocyte size.

Method for the determination of an adequate sample size for mFPA

From H&E stained sections of 5 patients with a range of mFPA, (3-43%), 50 non-overlapping images were taken at x20 OM (Zeiss Axioskop 40 with a Zeiss IcC5 camera). Using the same script described for the determination of sample adequacy of hepatocyte size the mFPA results from the 50 images per biopsy were analysed for sample sizes from 1-50. The plots show that a sample size of approximately 21 non-overlapping images taken at x20 OM is representative of the biopsy mFPA. Therefore, to assess mFPA of a biopsy, 21 randomly selected non-overlapping areas were imaged at x20 objective magnification (OM). Altogether 2100 at x20 OM were taken.

Supplementary Figure S2. A scatter plot showing the proportion of samples that were within 3% measured fat proportionate area (mFPA) of the reference value for each of the sample sizes from 1-50 fields at x20 objective magnification (OM).

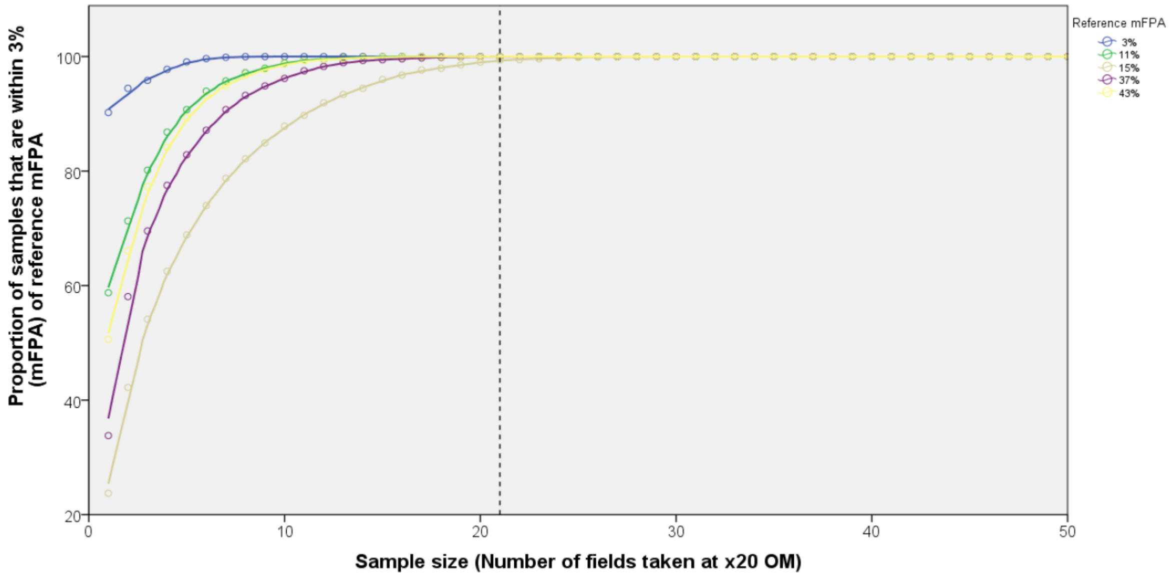

99.35-100% of samples were within 3% mFPA of the reference value when 21 fields taken at x20 OM were randomly selected from the each biopsy. Therefore to establish a protocol for mFPA for images taken at x20 OM we randomly selected 21 fields at x20 OM from each biopsy.

Supplementary Figure S3. A box plot showing the distribution of GGT, bilirubin, Alb and ALP when the population is split into increments of mFPA.

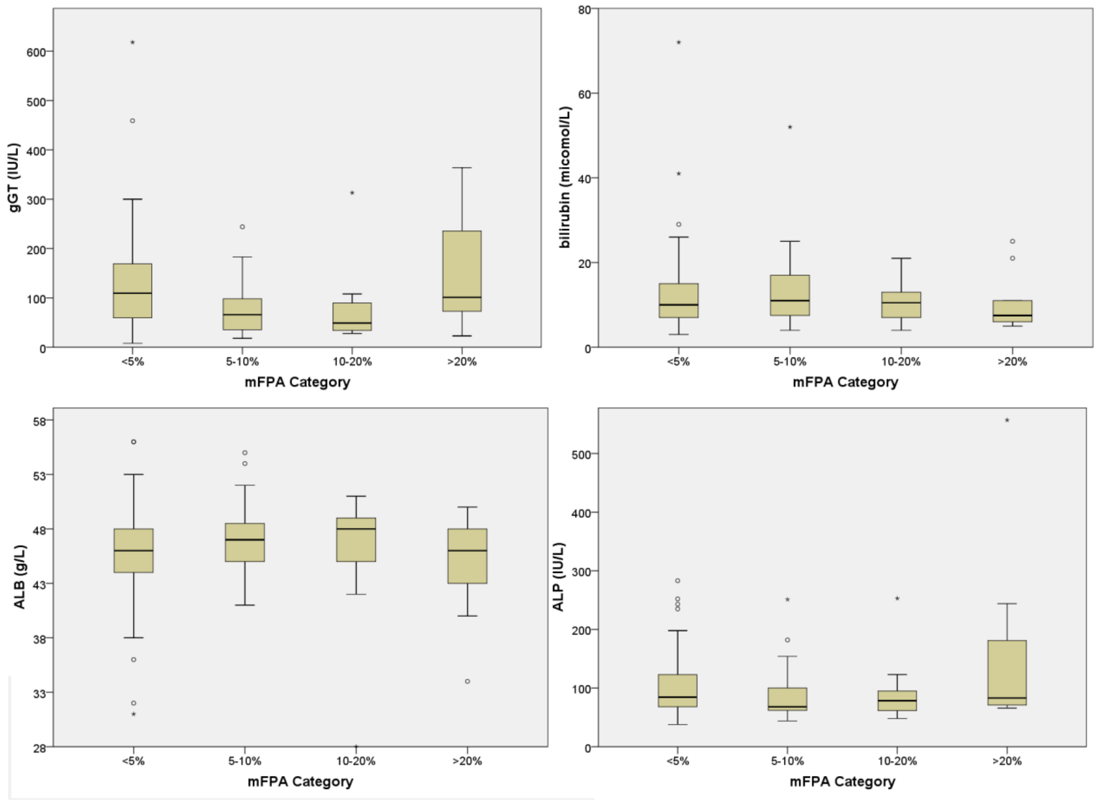

There is an overall significant difference in GGT across the categories (p=0.031). >5% mFPA showed significantly less GGT than <5% mFPA (p=0.020) but there are no other significant changes in GGT, Bilirubin, Albumin or ALP at 5% or 20% mFPA cut-off.

Reference List

1. Hall,A.R., Tsochatzis,E., Morris,R., Burroughs,A.K., & Dhillon,A.P. Sample size requirement for digital image analysis of collagen proportionate area in cirrhotic livers. *Histopathology* **62**, 421-430 (2013).
